# Investigation of the optimal indocyanine green dose in real-time fluorescent cholangiography during laparoscopic cholecystectomy with an ultra-high-definition 4K fluorescent system: a randomized controlled trial

**DOI:** 10.1007/s13304-023-01557-w

**Published:** 2023-06-14

**Authors:** Hui Liu, Jiao Kuang, Yujie Xu, Tianyang Li, Peilin Li, Zisheng Huang, Shuai Zhang, Jiefeng Weng, Yueyuan Lai, Zhaofeng Wu, Fan Lin, Weili Gu, Yu Huang

**Affiliations:** 1 Department of Hepatobiliary Pancreatic Surgery, Guangzhou First People’s Hospital, No.1 Panfu Road, Yuexiu District, Guangzhou, 510180 Guangdong People’s Republic of China; 2 Guangzhou Digestive Disease Center, Guangzhou First People’s Hospital, Guangzhou, China

**Keywords:** Indocyanine green, Laparoscopic cholecystectomy, Fluorescent cholangiography, Randomized controlled trial, 4K, OptoMedic

## Abstract

This study aimed to investigate the indocyanine green (ICG) dose in real‐time fluorescent cholangiography during laparoscopic cholecystectomy (LC) with a 4K fluorescent system. A randomized controlled clinical trial was conducted in patients who underwent LC for treatment of cholelithiasis. Using the OptoMedic 4K fluorescent endoscopic system, we compared four different doses of ICG (1, 10, 25, and 100 µg) administered intravenously within 30 min preoperatively and evaluated the fluorescence intensity (FI) of the common bile duct and liver background and the bile-to-liver ratio (BLR) of the FI at three timepoints: before surgical dissection of the cystohepatic triangle, before clipping the cystic duct, and before closure. Forty patients were randomized into four groups, and 33 patients were fully analyzed, with 10 patients in Group A (1 µg), 7 patients in Group B (10 µg), 9 patients in Group C (25 µg), and 7 patients in Group D (100 µg). The preoperative baseline characteristics were compared among groups (*p* > 0.05). Group A showed no or minimal FI in the bile duct and liver background, while Group D showed extremely high FIs in the bile duct and in the liver background at the three timepoints. Groups B and C presented with visible FI in the bile duct and low FI in the liver background. With increasing ICG doses, the FIs in the liver background and bile duct gradually increased at the three timepoints. The BLR, however, showed no increasing trend with an increasing ICG dose. A relatively high BLR on average was found in Group B, without a significant difference compared to the other groups (*p* > 0.05). An ICG dose ranging from 10 to 25 µg by intravenous administration within 30 min preoperatively was appropriate for real‐time fluorescent cholangiography in LC with a 4K fluorescent system. *Registration*: This study was registered in the Chinese Clinical Trial Registry (ChiCTR No: ChiCTR2200064726).

**Supplementary Information:**

The online version contains supplementary material available at 10.1007/s13304-023-01557-w.

## Introduction

With several decades of development in China, laparoscopic hepatobiliary surgery has been widely accepted and popularized. In recent years, a series of novel technologies, such as three-dimensional (3D) high-definition laparoscopic systems, ultra-high-definition (4K) laparoscopy, near-infrared fluorescence (NIRF) imaging techniques, and telesurgery with 5G wireless system, have injected new vitality into laparoscopic surgery in a new era [1]. A recently published study showed that the performance of 4K imaging systems in laparoscopic cholecystectomy (LC) was comparable to that of 3D laparoscopic systems, and no significant differences were found in operative time or error scores with these two systems [2]. 4K technology was reported to significantly improve the surgeon’s field of view resolution, enhance the fineness of the image, present clearer and more realistic surgical images on the screen, and improve the operator’s recognition of different tissues and the operational experience in the surgical field. However, to our knowledge, no studies have applied the NIRF imaging technique with indocyanine green (ICG) combined with the 4K fluorescent system for the purpose of real‐time fluorescent cholangiography during LC.

Fluorescent intraoperative cholangiography using NIRF and ICG was introduced during LC [3–5] to provide real-time visualization of the extrahepatic biliary tract, thereby playing a navigational role in hepatobiliary surgery [6]. It is thought that ICG-NIRF may be a promising tool to increase safety during LC [7]. Previously, we investigated the optimal ICG dose in real‐time fluorescent cholangiography in LC with a two-dimensional high-definition fluorescent system [8]. By intravenously injecting ICG within 30 min preoperatively, we showed that administration of 0.1 mg of ICG was statistically superior for fluorescent cholangiography of extrahepatic biliary structures before dissection and clipping of the cystohepatic triangle [8]. However, by applying the same ICG dose (0.1 mg of ICG) with the 4K system, the fluorescent effect was unsatisfactory because of the high background in the liver. In the present clinical study, therefore, we investigated the optimal ICG dose suitable for use with the 4K fluorescent system for real‐time fluorescent cholangiography in LC. The data showed that an ICG dose ranging from 10 to 25 µg administered intravenously within 30 min preoperatively was appropriate for real‐time fluorescent cholangiography during LC with a 4K fluorescent system.

## Materials and methods

### Patients

Patients with cholecystolithiasis and cholecystitis who were admitted to our department for treatment with LC or common bile duct (CBD) exploration (CBDE) were evaluated for enrollment. Inclusion criteria were as follows: patients aged ≥ 18 years and undergoing LC or LC + CBDE, without signs of liver cirrhosis in computed tomography or ultrasound imaging. Exclusion criteria were as follows: allergies to iodides, iodine dyes, or ICG. All patients provided informed, written consent.

### Study design

A randomized controlled trial was conducted. The primary outcome was the fluorescence intensity (FI) of the common bile duct and liver at three timepoints: before surgical dissection of the cystohepatic triangle, before clipping the cystic duct, and before closure or before CBDE. The second outcome was the cholangiography effect, which was analyzed by the bile duct-to-liver ratio (BLR). Patients were randomly allocated for preoperative injection with one of the four doses of ICG (Group A: 1 µg; Group B: 10 µg; Group C: 25 µg; and Group D: 100 µg). This study was approved by the Research Ethics Committee of the Guangzhou First People’s Hospital (approval NO: B202203101) and complied with the requirements of the Declaration of Helsinki. This study was registered in the Chinese Clinical Trial Registry (ChiCTR No: ChiCTR2200064726).

### Imaging system

The 4K fluorescent system equipped with an NIRF imaging system (FloNavi 214K Series) was provided by Guangdong OptoMedic Technologies Inc, Guangzhou, China (web link: http://www.optomedic.com). This 4K system has four different imaging modalities, a 4K white light mode, a 4K standard fluorescence mode, a color scale fluorescence mode, and a multi-display mode with a grayscale image, and the modality could be changed during surgery (Figure S1).

### Sample size

G*Power [9] was used to estimate the sample size as previously reported [8]. Based on a previous study, the baseline rate for bile duct detection with ICG was estimated as 70% [8]. We therefore set the effect size f at 70%, the α-error at 0.05, the power (1 − β) at 0.95, and had four different groups. Finally, 40 patients were included in our trial, for which the critical *F* value was 2.866 and the actual power was 0.954 (Figure S2).

### Randomization

The randomization was performed as previously reported [8]. Briefly, the included patients were serially numbered from 01 to 40 according to the order of admission. Then, these 40 patients were randomly divided into four groups, with an allocation ratio of 1:1:1:1, using a randomization sequence generated by SPSS 26.0. Based on the results, the patients were randomly assigned to groups A, B, C, and D. A double-blinded setting was used. The serially numbered envelope containing dose and group information was matched to the recruited patient before surgery.

### Intervention

ICG was intravenously administered 30 min before surgery as previously reported [8]. Specifically, ICG was injected after the patient’s anesthetization, and any adverse reactions were recorded. We recorded the first images when the cystohepatic triangle was first visualized. We recorded the second images after dissecting the surrounding tissues of the gallbladder to permit the cholangiography of the cystic duct, common hepatic duct, and CBD, but before gall bladder resection, immediately before clipping. Finally, we obtained the last images before closing, or before starting exploration of the CBD when necessary. For each time point, four images in each of the four different imaging modalities were collected.

### FI measurements

The FI of the CBD and the liver was measured using ImageJ as previously reported [8]. The grayscale images from the multi-display mode were used. The regions of interest (ROIs) in the CBD were selected as the ICG areas, and the ROIs in the liver background were selected as the ICG areas present in the liver. The mean ROIs in the CBD ranged from 3000 to 6,500, and the ROIs in the liver ranged from 6000 to 19,500. The BLR was calculated as follows: BLR = FI_bile-duct_/FI_liver_.

### Statistical analysis

Data are presented as the mean ± standard deviation (SD) or actual number of cases. A two-sided *p* value < 0.05 was considered statistically significant. For intergroup comparisons of continuous variables, analysis of variance (ANOVA) was conducted. Statistical analysis was performed using SPSS 26.0, and the figures were generated with GraphPad Prism.

## Results

### Patients

**Fig. 1:**
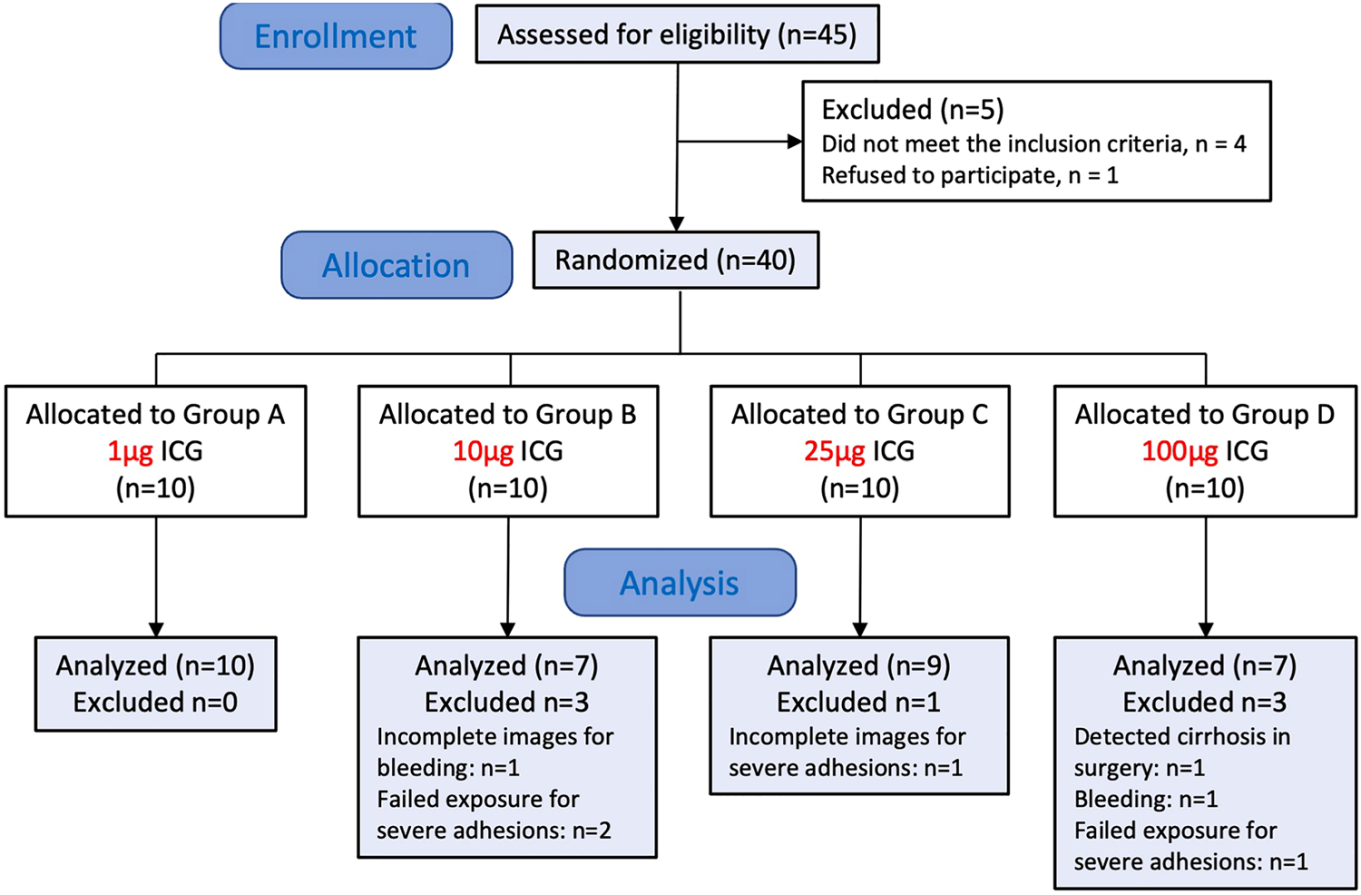
Flowchart of the study

Forty-five patients were assessed for eligibility. Five patients were excluded in which four patients did not meet the inclusion criteria and one patient refused to participate (Fig. 1). Forty patients were randomized, of which 33 patients were included for analysis, with 10 patients in Group A, 7 patients in Group B, 9 patients in Group C, and 7 patients in Group D (Fig. 1). In Group B, three patients were excluded for incomplete images as bleeding in one case and failed exposure as severe adhesions in two cases. In Group C, one case was excluded for incomplete images for severe adhesions. In Group D, three patients were excluded for liver cirrhosis detected during surgery in one case, failed exposure as severe adhesions in one case and bleeding in one case (Fig. 1). There were no differences in preoperative patient characteristics, diagnoses for surgery, or preoperative levels of aspartate aminotransferase, alanine aminotransferase, total bilirubin, or direct bilirubin, among groups (Table 1). There were no significant intergroup differences in operation time or postoperative hospitalization duration (Table 1).

**Table 1:** Baseline characteristics of the study patients according to the treatment groups

	Group A (*n* = 10)	Group B (*n* = 7)	Group C (*n* = 9)	Group D (*n* = 7)	*F *value	*p* value
Age (years)	53.5 ± 17.7	64.6 ± 17.9	57.7 ± 11.7	55.6 ± 13.1	0.66	0.58
Gender (M/F)	6/4	1/6	3/6	2/5	–	–
BMI	24.62 ± 1.98	24.466 ± 2.11	25.22 ± 2.28	25.13 ± 2.42	0.27	0.84
Diagnosis					–	–
Cholecystoli thiasis	10	7	9	7		
Cholecystitis	10	7	9	7		
Thickness of GB (< = 3 µm/ > 3 µm)	1/9	2/5	3/6	0/7	–	–
History of abdominal surgery	1	0	2	2	–	–
Preoperative AST (U/L)	141.5 ± 243.6	83.4 ± 82.8	41.8 ± 41.6	113.3 ± 114.1	0.65	0.59
Preoperative ALT (U/L)	225.8 ± 361.3	132.4 ± 127.0	61.4 ± 82.2	272.7 ± 211.9	1.19	0.33
Preoperative TBiL (µmol/L)	31.0 ± 25.1	29.8 ± 39.1	28.6 ± 29.5	21.8 ± 9.6	0.15	0.93
Preoperative DBiL (µmol/L)	13.9 ± 17.8	13.6 ± 25.3	12.4 ± 17.1	7.6 ± 5.2	0.17	0.91
Surgery (LC/LC + CBDE)	3/7	3/4	5/4	4/3	–	–
Operation time (min)	110.1 ± 39.4	115.0 ± 26.3	82.4 ± 27.6	84.4 ± 25.6	2.11	0.12
Postoperative hospitalization duration (d)	3.7 ± 2.4	5.4 ± 4.8	3.1 ± 1.4	3.4 ± 0.9	0.96	0.43

Data were presented as Mean ± S.D, or as case number

*LC* Laparoscopic cholecystectomy, *CBDE* Common bile duct exploration, *AST* Aspartate aminotransferase, *ALT* Alanine aminotransferase, *TBiL* Total bilirubin, *DBil* Direct bilirubin, *SD* Standard deviation

### Cholangiography

**Fig. 2:**
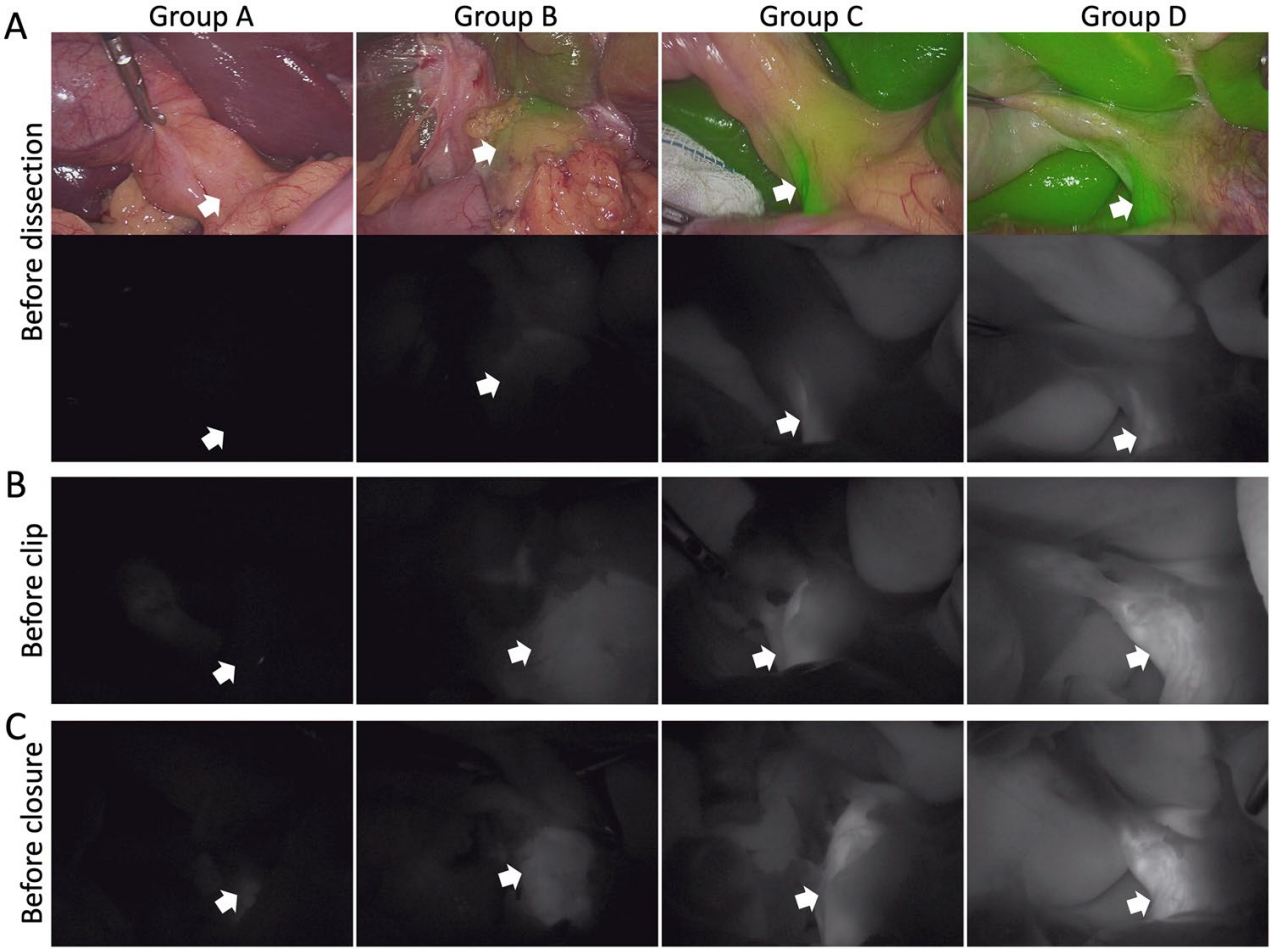
Representative graphs of the three time points. **A** shows the fluorescent images and grayscale image before dissection of the cystohepatic triangle; **B** shows the grayscale image before clipping the cystic duct; **C** shows the grayscale image before closure. The white arrow shows the common bile duct

As shown in Fig. 2, Group A showed no or a few fluorescent areas in the bile duct and no liver background, demonstrating that this low dose of ICG (i.e., 1 µg) was insufficient for cholangiography for the duration of the surgery. In contrast, Group D showed extremely high intensity fluorescent areas in the bile duct with a high liver background at the three timepoints, demonstrating that this high dose of ICG (i.e., 100 µg) was excessive for cholangiography. Group B showed visible fluorescence in the bile duct region without a liver background, and Group C presented with clear fluorescence in the bile duct with a moderate liver background.

### FI and BLR outcomes

**Fig. 3:**
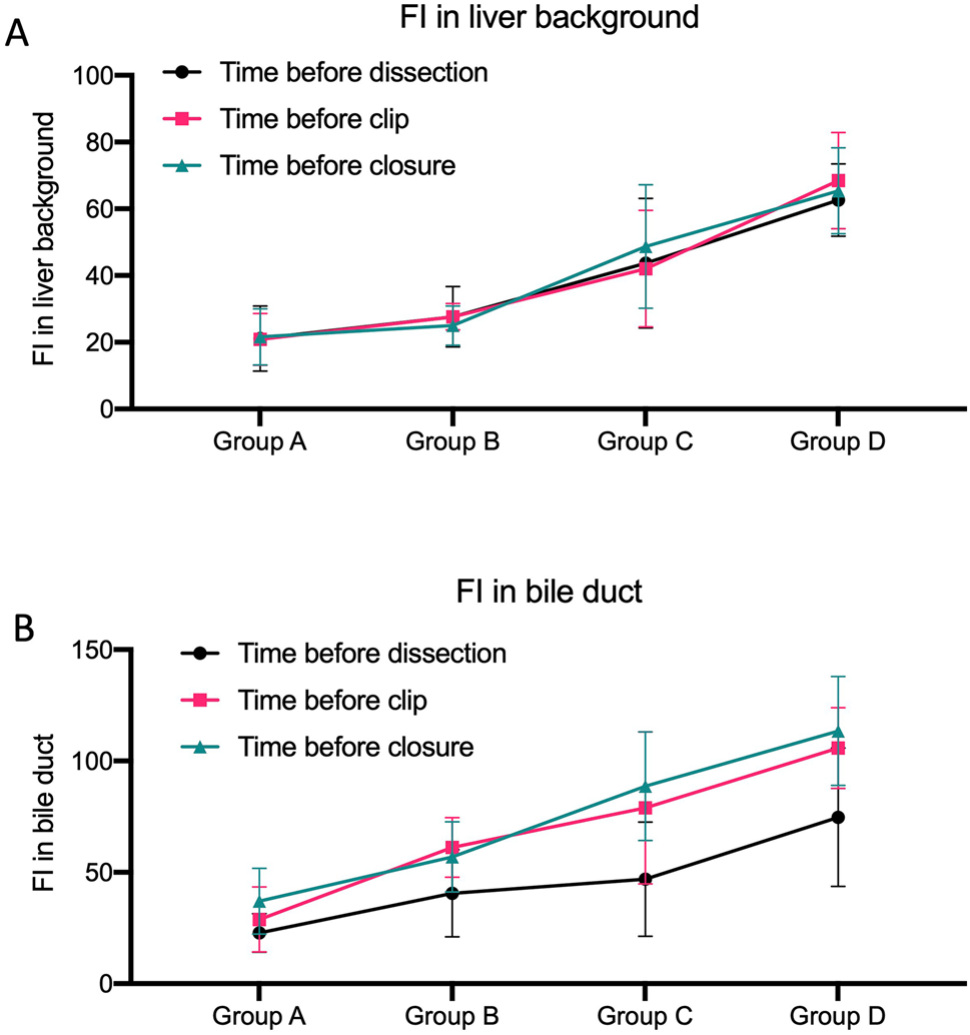
Levels of the fluorescence intensity (FI) in the liver background and bile duct. **A** shows the FIs in the liver background at the three measurement time points: before surgical dissection of the cystohepatic triangle (black line), before clipping the cystic duct (red line), and before closure (blue line); **B** shows the FIs in the bile duct before surgical dissection of the cystohepatic triangle (black line), before clipping the cystic duct (red line), and before closure (blue line). FI fluorescent intensity

**Fig. 4:**
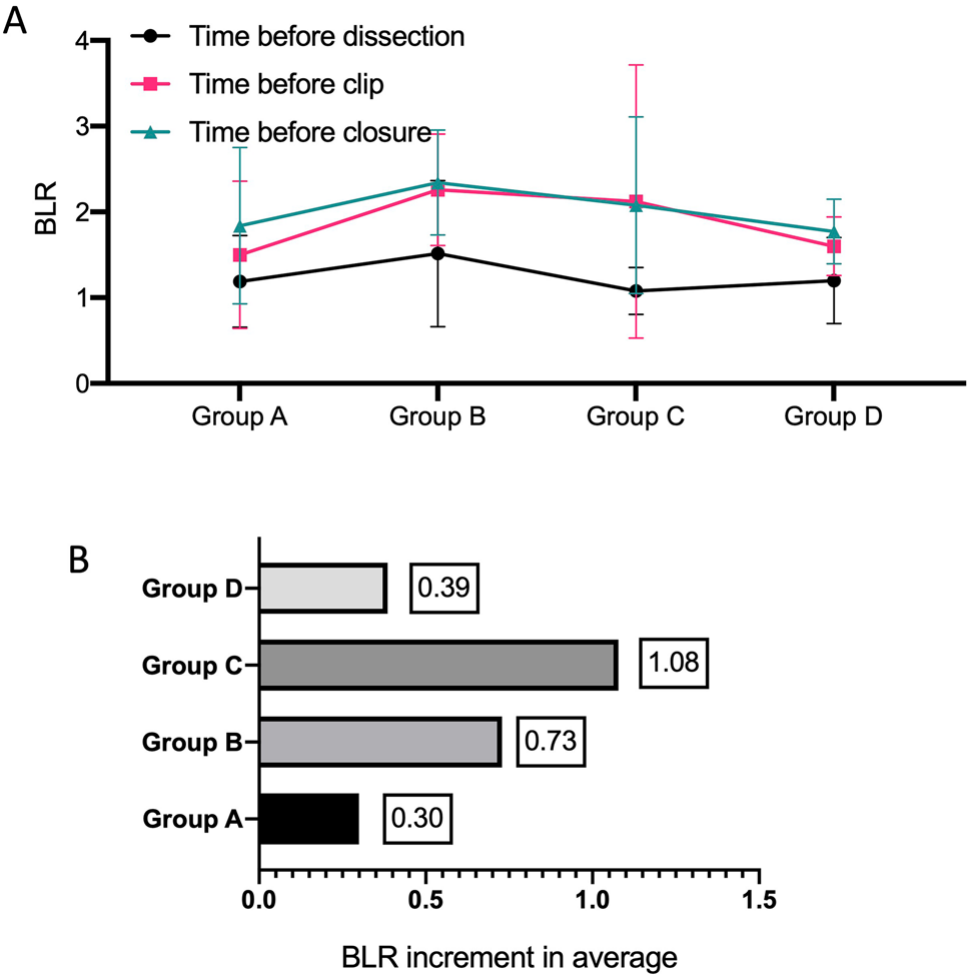
Bile-to-liver ratio (BLR) and the BLR increment at the three time points. **A** shows the BLRs before surgical dissection of the cystohepatic triangle (black line), before clipping the cystic duct (red line), and before closure (blue line). **B** shows the average BLR increment from the point of surgical dissection of the cystohepatic triangle to clipping the cystic duct

With increasing ICG doses, i.e., from Group A to Group D, the FIs in the liver background (Fig. 3A) and bile duct (Fig. 3B) gradually increased at all three timepoints. For all groups, the FIs in the liver background showed no decrease or increase among the three timepoints, demonstrating a constant fluorescence in the liver background (Fig. 3A). In the bile ducts, the fluorescence was greater prior to clipping and before closure compared to the fluorescence before dissection (Fig. 3B), demonstrating an increasing fluorescence in the bile duct during surgery. The BLR, however, showed no increasing trend with an increasing ICG dose (Fig. 4A). Group B had a relatively high BLR on average, but without a significant difference compared to the other groups (*p* > 0.05). Accordingly, Group B and Group C showed a relatively high BLR increment on average (0.73 in Group B and 1.08 in Group C vs. 0.3 in Group A and 0.39 in Group D) from the timepoint before dissection to the timepoint before clipping (Fig. 4B).

## Discussion

With the clinical application of novel technologies in recent years, many standard procedures should be updated accordingly. In this study, a clinical trial was designed to investigate the optimal ICG dose suitable for use with a 4K fluorescent system for real‐time fluorescent cholangiography in LC. The data showed that, with a 4K fluorescent system, an ICG dose ranging from 10 to 25 µg by intravenous administration within 30 min preoperatively was appropriate for real‐time fluorescent cholangiography during LC. Compared to the dose of ICG used with 2D high-definition fluorescent systems [8], the ICG dose was lower in the 4K fluorescent system (10–25 µg in the 4K system compared to 100 µg in the 2D system). This may contribute to the high resolution of the 4K system. Furthermore, the lower concentration of ICG in the body confers a lower risk of injury to the liver. One of the greatest contribution of this study is that our data provide surgeons with an appropriate ICG dose for real‐time fluorescent cholangiography in LC with an ultra-high-definition 4K fluorescent system.

Dose, time interval, and the administration route of ICG are the three key factors that affect the cholangiographic effect during LC [10–12]. The administration route can be intravenous and intrabiliary [13–16]. Both routes offer images of biliary visualization in real time, which helps surgeons to select the optimal point to transect the cystic duct or hepatic duct [14]. In this study, we chose the intravenous route for its easy implementation without bile leak-out, which is an advantage of the intravenous route compared to the intrabiliary route. The time interval of ICG injection varied from 1 to 24 h in previously reported studies [10]. However, in this study, ICG was administered 30 min before surgery as we believe this time interval was a more clinically practical time interval with high controllability [8]. Usually, 5 min is required for nerve block anesthesia, 5 min for urinary catheterization, about 3–5 min for surgeon hand washing, 5 min for disinfection of the surgical area, 5 min for preparation of the imaging system, camera sensor, and light source, and 1–3 min for the patient information checklist before surgery. Therefore, 30 min before surgery was selected for the ICG injection. Therefore, ICG administration could be performed between intubation and the nerve block anesthesia or urinary catheterization, as previously reported [8]. 

Because of the high sensitivity of the 4K system, a lower dose of ICG than used with the HD system was proposed. The verified ICG dose (0.1 mg of ICG) used with the HD system was found unsatisfactory in the 4K system, as it showed a high background in the liver. Therefore, four different ICG doses (1, 10, 25, and 100 µg) were evaluated in this study. The data demonstrated that an ICG dose ranging from 10 to 25 µg was appropriate for real‐time fluorescent cholangiography in LC. The very-low dosage of ICG in our trail was extremely different from the previously reported studies. Most of those studies utilized dosage of 2.5 mg [17–21], while small size of reported studies used the 5 mg or 10 mg [22, 23]. In 2015, Kawaguchi and colleagues reported their experience of cholangiography using a lower of 0.025 mg ICG after intubation [24]. However, they used this low dosage for ICG to perform a laparoscopic liver resection with Olympus laparoscopic imaging system. Therefore, the available imaging systems or technologies may be regarded as the fourth key factors affecting surgical cholangiographic effect. This trial may help to further our knowledges on update the application of fluorescent cholangiography with different imaging systems.

According to the data of our trial, the FIs in the liver background and bile duct were ICG dose-independent, consistent with previously reported research [6, 8]. The study showed that the FIs in the liver background and bile duct gradually increased with increasing ICG doses. However, the BLR, a useful index for the cholangiographic effect during the surgical course [3, 25, 26], showed no increasing trend with increasing ICG dose. This implied that the appropriate dose of ICG in the 4K system may be a range, and that may be caused by the high sensitivity of the 4K system. To describe the changing trend of the BLR, we introduced a new parameter, the BLR increment. As shown in Fig. 4B, Group C (25 µg ICG group) showed a relatively high BLR increment on average from the timepoint before dissection to the timepoint before clipping. From this point of view, 25 µg of ICG was found to be appropriate for the real‐time fluorescent cholangiography in LC.

This study has several limitations. One of the limitations was that no follow-up data were analyzed. However, it is acceptable for this study, because we focused on fluorescent cholangiography during the surgery for navigational purposes. Furthermore, the enrolled sample size is small. We did not take the body weight factor into account in this single-center study for no significant differences of the body mass index among the four groups (Table 1). Future multicenter studies with large sample size and including body weight factor remain needed. In addition, only patients with relatively normal liver function without liver cirrhosis or without complete biliary obstruction were included in the study. The allocation and distribution of ICG are influenced by hepatocyte function and biliary obstruction. Therefore, the cholangiographic effect of ICG may differ in patients with or without liver cirrhosis or complete biliary obstruction. Detailed information is needed for patients with liver cirrhosis or complete biliary obstruction. A clinical comparison between normal and abnormal levels of total bilirubin would be beneficial in future studies of patients presenting with biliary obstruction.

## Conclusions

In conclusion, with a 4K fluorescent system, an ICG dose ranging from 10 to 25 µg by intravenous administration within 30 min preoperatively was found to be appropriate for real‐time fluorescent cholangiography during LC.

### Supplementary Information

Below is the link to the electronic supplementary material.

Supplementary file1 (JPG 781 KB)

Supplementary file2 (JPG 611 KB)

## Data Availability

All data generated or analyzed during this study are included in this published article.

## Abbreviations

3D: Three-dimensional
4K: Ultra-high-definition
BLR: Bile-to-liver ratio
CBD: Common bile duct
CBDE: Common bile duct exploration
FI: Fluorescence intensity
ICG: Indocyanine green
LC: Laparoscopic cholecystectomy
NIRF: Near-infrared fluorescence
