# Supplementary figures and images for: Investigation of the optimal indocyanine green dose in real-time fluorescent cholangiography during laparoscopic cholecystectomy with an ultra-high-definition 4K fluorescent system: a randomized controlled trial
Source: Updates Surg. 2023 Jun 14;75(7):1903–10. doi: 10.1007/s13304-023-01557-w (PMC10543949; doi:10.1007/s13304-023-01557-w)

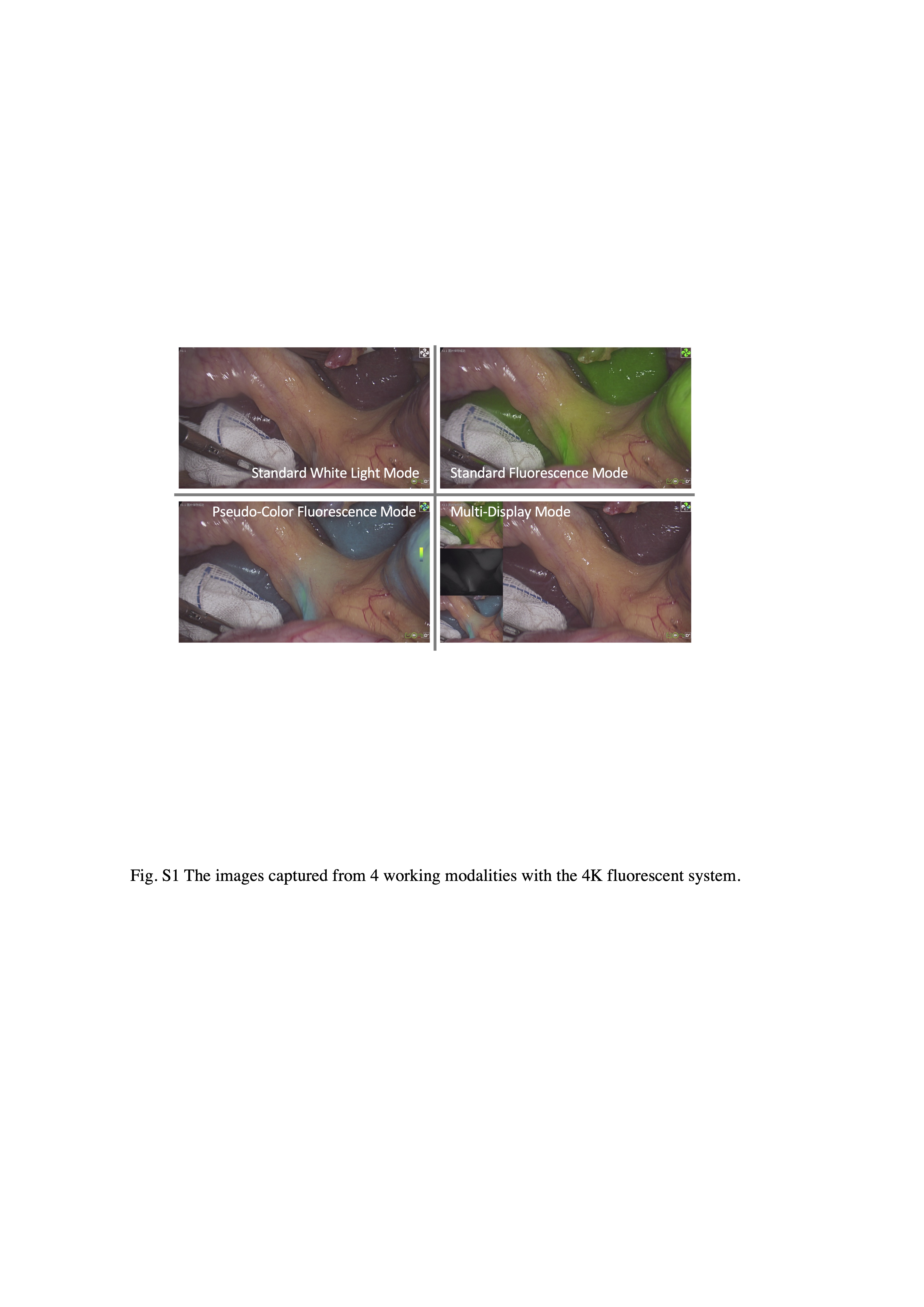

Supplement: Supplementary file 1 — Supplementary file1 (JPG 781 KB) [file 13304_2023_1557_MOESM1_ESM.jpg]

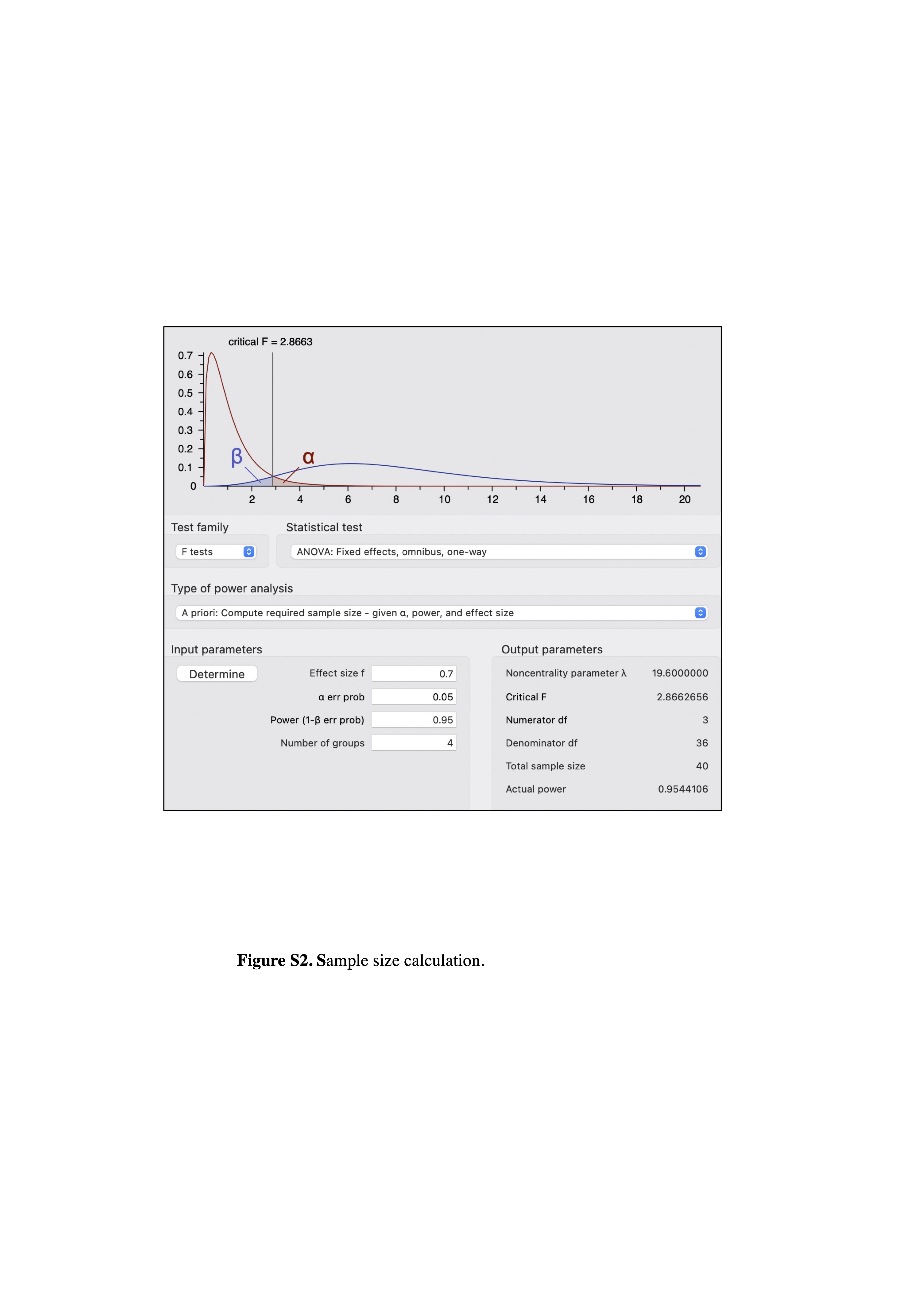

Supplement: Supplementary file 2 — Supplementary file2 (JPG 611 KB) [file 13304_2023_1557_MOESM2_ESM.jpg]
